# The genotype‐phenotype landscape of an allosteric protein

**DOI:** 10.15252/msb.202110847

**Published:** 2021-12-16

**Authors:** Drew S Tack, Peter D Tonner, Abe Pressman, Nathan D Olson, Sasha F Levy, Eugenia F Romantseva, Nina Alperovich, Olga Vasilyeva, David Ross

**Correction to:** *Mol Syst Biol* (2021) 17: e10179. DOI: 10.15252/msb.202010179 | Published online 30 March 2021

In this study, the authors stated: “To our knowledge, this is the first identification of single‐protein genetic sensors with band‐stop dose‐response curves”. After the publication of the Tack *et al* (2021) study and while preparing a related manuscript, they found some early publications describing LacI variants with “band‐stop” dose–response curves. In those earlier papers (from the 1960s and 1970s), what Tack *et al* (2021) refer to as band‐stop variants were denoted as the “reversed” or “*i* ^r^” phenotype. While preparing the Tack *et al* (2021) study, they mistakenly thought that “reversed” was synonymous with “inverted” (a term also used in other recent studies). As such, despite being aware that “reversed” variants had been observed before, they did not realize that they were similar to what they refer to as “band‐stop” variants.

The earlier publications describing reversed (i.e., band‐stop) LacI variants include the following: a study of temperature‐sensitive variants (Sadler & Novick, 1965); studies of the LacI X86 mutant, a reversed variant that is not temperature‐sensitive (Chamness & Willson, 1970; Jobe & Bourgeois, 1972); studies of variants that bind to DNA operator more tightly than the wild‐type (Betz & Sadler, 1976; Schmitz *et al*, 1978); and studies of a large number of mutations, including X86 and other reversed variants (Miller & Schmeissner, 1979; Miller *et al*, 1979).

Besides the sentence quoted above, this mistake does not impact the findings of the study. The authors apologize for this error.
