# Supplementary material for: The genotype‐phenotype landscape of an allosteric protein
Source: Mol Syst Biol. 2021 Mar 30;17(3):e10179. doi: 10.15252/msb.202010179 (PMC8009258; doi:10.15252/msb.202010179)
Supplement: Supplementary file 1 — Appendix [file MSB-17-e10179-s005.pdf]

Appendix for

The genotype-phenotype landscape of an allosteric protein

Drew S. Tack, Peter D. Tonner, Abe Pressman, Nathanael D. Olson, Sasha F. Levy, Eugenia F. Romantseva, Nina Alperovich, Olga Vasilyeva, David Ross*

Correspondence to: david.ross@nist.gov

Table of Contents

Appendix Figure S1 - Plasmid maps used for the library-scale and verification measurements. 3
Appendix Figure S2 - Comparison of fluorescence distribution of the LacI library before and after fluorescence activated cell sorting (FACS) sorting. 4
Appendix Figure S3 - Diversity of phenotypes in the LacI library. 5
Appendix Figure S4 - Amino acid substitution count heat map. 6
Appendix Figure S5 - Example data for fitness and dose-response curves for normal phenotype LacI variants. 7
Appendix Figure S6 - Example data for fitness and dose-response curves for inverted phenotype LacI variants. 8
Appendix Figure S7 - Example data for fitness and dose-response curves for band-stop phenotype LacI variants. 9
Appendix Figure S8 - Example data for fitness and dose-response curves for band-pass phenotype LacI variants. 10
Appendix Figure S9 - Distributions of Hill equation parameters measured for the full library and the wild-type variants. 11
Appendix Figure S10 - Calibration data for the determination of dose-response curves using barcode sequencing. 12
Appendix Figure S11 - Gene expression measurement uncertainty for library-scale measurement of LacI dose-response curves. 13
Appendix Figure S12 - Deep neural network (DNN) model. 14
Appendix Figure S13 - Prediction of LacI variant phenotypes with recurrent deep neural network (DNN) model. 15
Appendix Figure S14 DNN model prediction uncertainty and root-mean-square error (RMSE). 16
Appendix Figure S15 - Comparison between the DNN model root-mean-square error (RMSE) and the measurement uncertainty for single mutants. 17
Appendix Figure S16 - Measured vs. predicted values from DNN model for single amino acid substitutions. 18
Appendix Figure S17 - Heat map of single substitution effects on G_0. 19
Appendix Figure S18 - Heat map of single substitution effects on G_∞. 20
Appendix Figure S19 - Heat map of single substitution effects on EC_{50}. 21
Appendix Figure S20 - Multiparametric impact of amino acid substitutions on allosteric function of LacI. 22
Appendix Figure S21 - Comparison between log-additive prediction and measurement for double-substitution variants for G_0 and G_∞. 23
Appendix Figure S22 Nearest neighbor distance histograms for inverted and band-stop LacI variants. 24

Appendix Figure S23 - The band-stop phenotype emerges from the combination of two amino acid substitutions. 25
Appendix Figure S24 - Measurement of OD_{600} vs. time for *E. coli* growth during initial, 12-hour incubation in automated culture protocol. 26
Appendix Figure S25 - Measurement of OD_{600} vs. time for *E. coli* in Growth Plates 1-4. 27
Appendix Figure S26 - Example of OD_{600} vs. time measurement for a full *E. coli* growth curve. 28
Appendix Figure S27 - Flow cytometry gating example. 29
Appendix Table S1 - Number of measured genotypes in library for different mutational distances from wild-type *lacI*. 30
Appendix Table S2 - LacI protein structural domains and features referred to in the manuscript. 31
Appendix Table S3 - Amino acid substitutions and structural domains or features associated with the inverted LacI phenotype. 32
Appendix Table S4 - Amino acid substitutions and structural domains or features associated with the band-stop LacI phenotype. 33
Appendix Table S5 - Cell growth in the 24 chemical environments used for library-scale measurement of LacI dose-response curves. 34
Appendix Table S6 - Regions extracted from long-read sequencing data for library-scale analysis. 35
Appendix Table S7 – PCR primers used during LacI library construction 36
Appendix Table S8 – Forward index primers for barcode amplification. 37
Appendix Table S9 - Reverse index primers for barcode amplification. 38
Appendix Table S10 - Primers sequences for the second PCR. 39
References 40

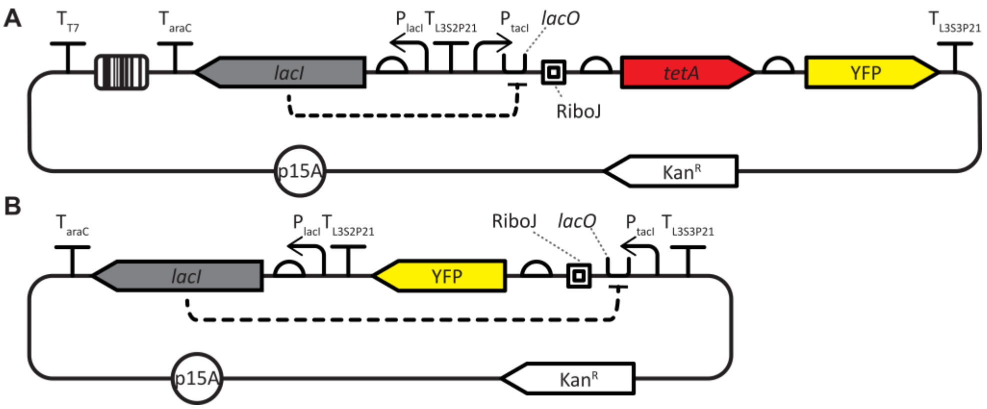

Appendix Figure S1 - Plasmid maps used for the library-scale and verification measurements.

Both plasmids contain the p15A origin of replication (p15A), kanamycin resistance gene (Kan^R), and *lacI* coding DNA sequence (*lacI*). In both plasmids, the encoded LacI protein transcriptionally regulates an output that was driven by the P_{tacI} promoter, *lacO* operator, and RiboJ transcriptional insulator.

A The library plasmid (pTY1) was used for measurement of the genotype and phenotype of the entire LacI library. The LacI library, including the coding DNA sequences of *lacI* variants and their corresponding barcodes, was cloned into pTY1. In pTY1, the LacI variants regulated the expression of a tetracycline resistance gene, *tetA*. Plasmid pTY1 also encoded yellow fluorescence protein (YFP), which was used for cloning purposes.

B The verification plasmid (pVER) was used to verify the dose-response curves of over 100 variants from the library. To verify the dose-response curve of a LacI variant, the coding DNA sequence for that variant was chemically synthesized and cloned into pVER, where the LacI variant regulated the expression of YFP. The dose-response curve was then measured using flow cytometry.

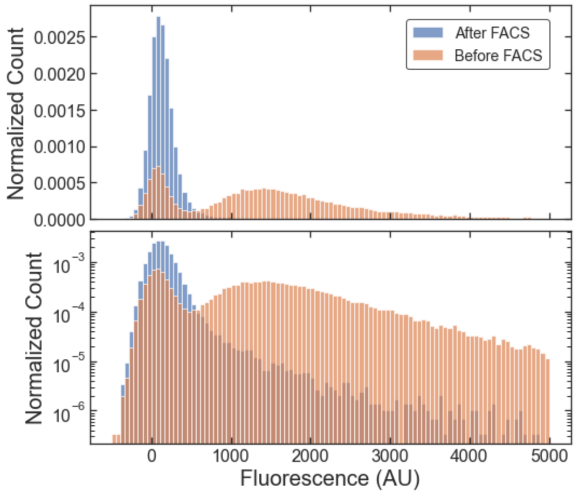

Appendix Figure S2 - Comparison of fluorescence distribution of the LacI library before and after fluorescence activated cell sorting (FACS) sorting.

To generate a library in which most of the LacI variants could function as allosteric repressors, FACS was used to select a portion of the library with low fluorescence in the absence of ligand. To allow comprehensive long-read sequencing of the library, the library size was further reduced by dilution of the FACS-selected library to create a population bottleneck of approximately 10^5 LacI variants. The orange histogram bars show the fluorescence distribution for the library before the FACS sorting. The blue histogram bars show the fluorescence distribution for the final library used for the library-scale measurements. The bottom plot shows the same histogram as the top plot with the y-axis on a logarithmic scale.

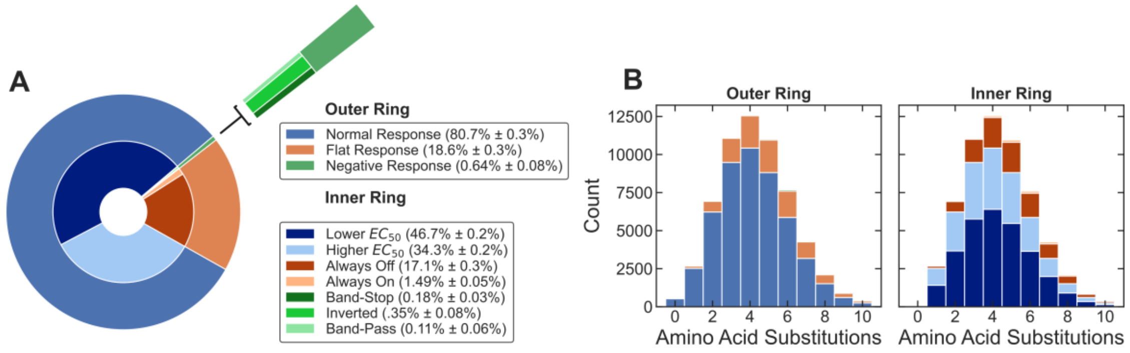

Appendix Figure S3 - Diversity of phenotypes in the LacI library.

A Relative abundance of the various LacI phenotypes found in the library. Variants with a "normal response" phenotype have dose-response curves qualitatively similar to the wild-type, with $G_0 < G_\infty$. Normal response variants include variants with EC_{50} lower than the wild-type value (between approximately 1 µmol/L and 100 µmol/L) and variants with EC_{50} higher than the wild-type value (between approximately 100 µmol/L and 2000 µmol/L). Variants with a "flat response" phenotype have flat dose-response curves with $G_0 \cong G_\infty$. Flat response variants include always-off variants ($G(0) < 0.25 \times G_{\infty,wt}$; i.e., the I^S phenotype from Markiewicz *et al.*, *Journal of Molecular Biology*, **240**, 421-433, 1994) and always-on variants ($G(0) > 0.25 \times G_{\infty,wt}$; i.e. the I^- phenotype from Markiewicz *et al.*). Variants with a "negative response" phenotype have dose-response curves with a negative slope, $\partial G/\partial L$, for some portion of the measured concentration range. Negative response variants include band-stop, inverted, and band-pass variants.

B Histogram of the number of variants with each LacI phenotype found in the library as a function of the number of amino acid substitutions.

The plots in (A) and (B) share the same legend. The outer ring of (A) and the left panel of (B) show the proportion of variants with dose-response curves that are normal, flat-response, or negative response. The inner ring of (A) and the right panel of (B) show more detailed descriptors of variant phenotypes.

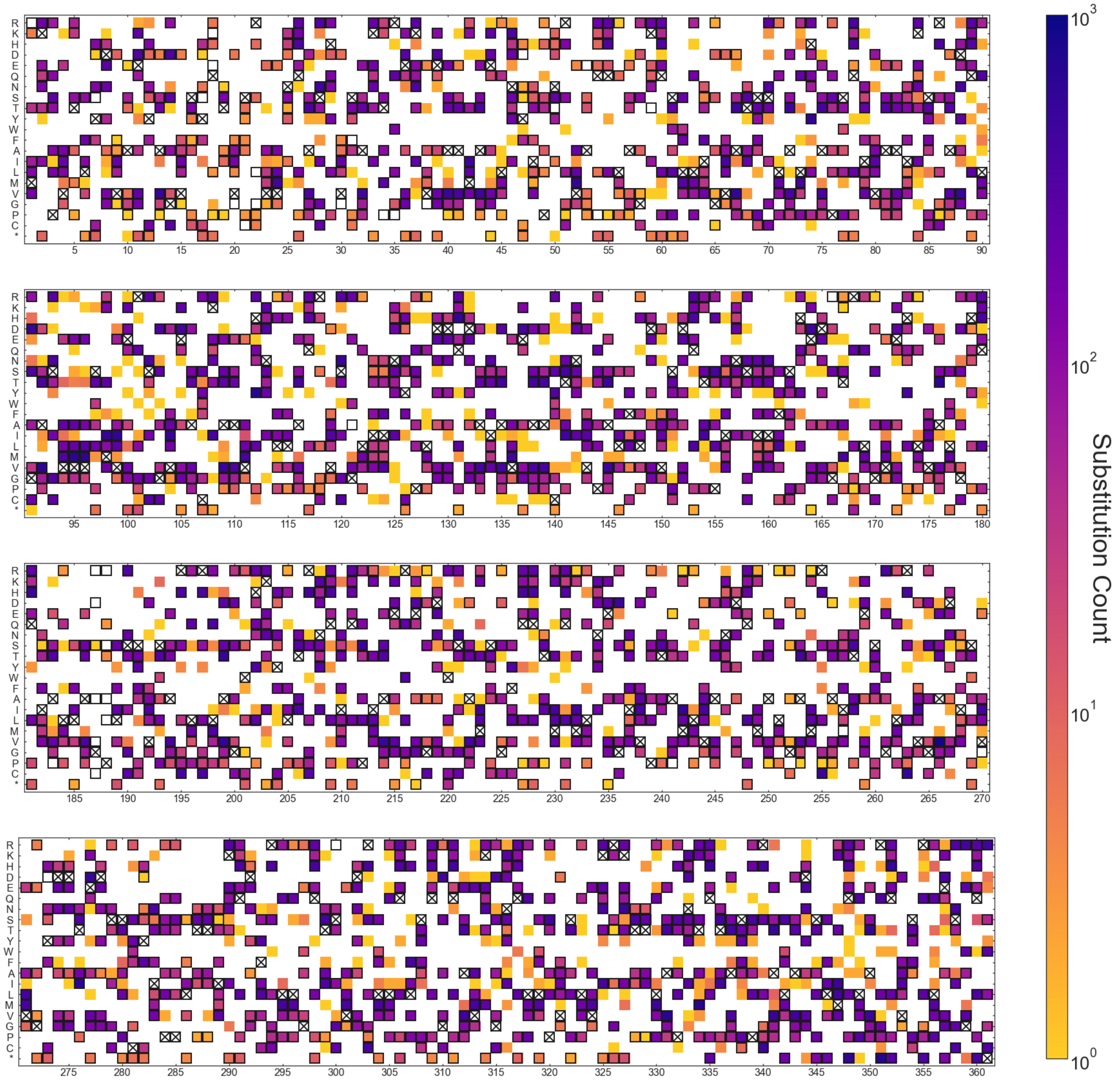

Appendix Figure S4 - Amino acid substitution count heat map.

The heat map indicates the number of times each possible amino acid substitution was observed across the entire LacI library, with the residue number as the x-axis and the possible amino acid substitutions as the y-axis. The wild-type amino acid at each residue is marked with an 'X'. SNP-accessible substitutions (possible via a single DNA base change per codon) are outlined with black squares. Substitutions that were not observed for any variant in the library are shown as white. Substitutions at residues 187 and 188 are under-represented because those codons contained a restriction site used during library and plasmid assembly. Most of the other SNP-accessible substitutions that were not observed were probably excluded by FACS selection during library preparation (see Appendix Figure S2 and Materials and Methods).

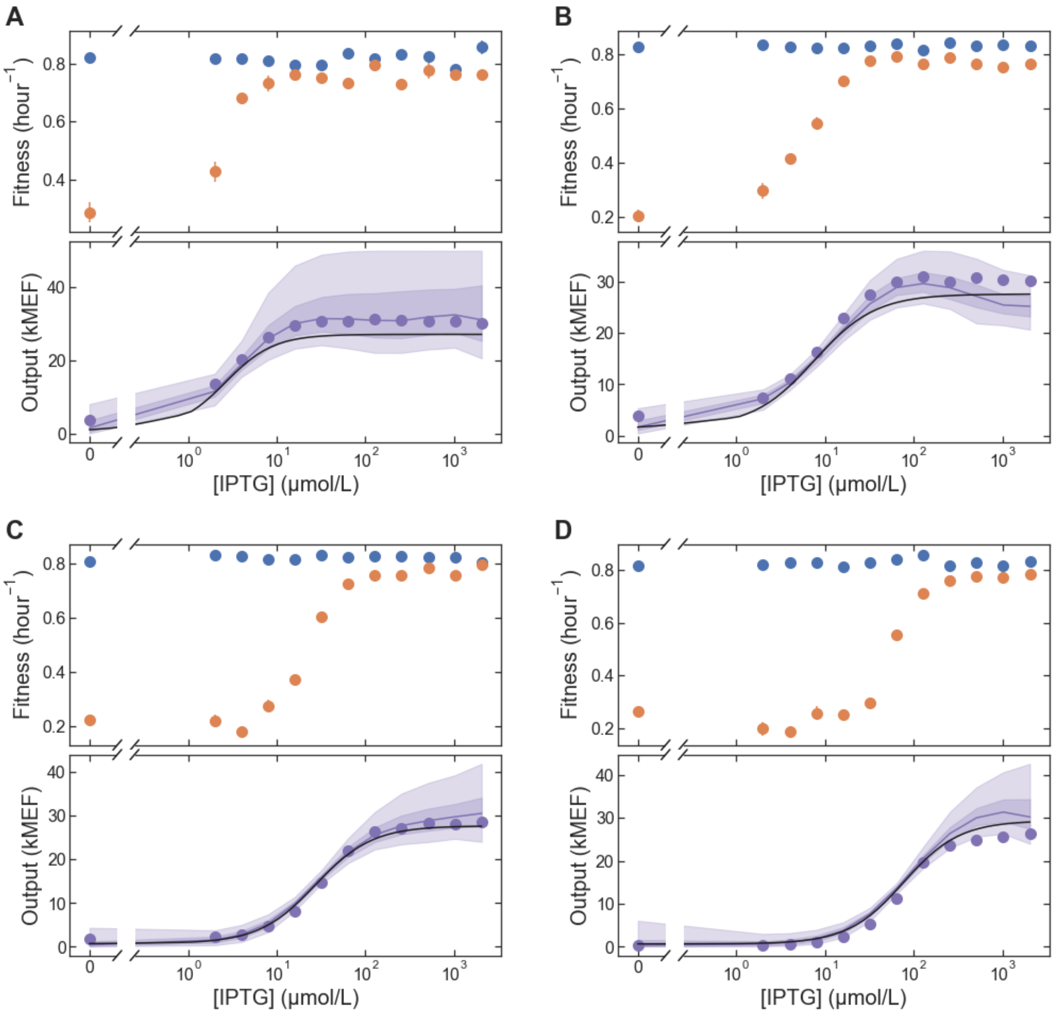

Appendix Figure S5 - Example data for fitness and dose-response curves for normal phenotype LacI variants.

Each pair of plots shows the fitness (top) and dose-response curve (bottom) for a different LacI variant. The fitness data is from the library-scale landscape measurement. The fitness without tetracycline is shown as blue points and the fitness with tetracycline is shown as orange points. In the dose-response plots, the black lines show the results from the library-scale measurement using the Bayesian Hill equation model. The purple lines and shaded regions show the results from the library-scale measurement using the Bayesian Gaussian process (GP) model, where the line is the median GP results and the shaded regions indicate 50% and 90% credible intervals. The plotted purple points show the dose-response curves from the flow cytometry verification measurements. Error bars indicate ± one standard deviation estimated from the least-squares fit (fitness, top plots) or the Bayesian posterior (output, bottom plots), and are often within the markers.

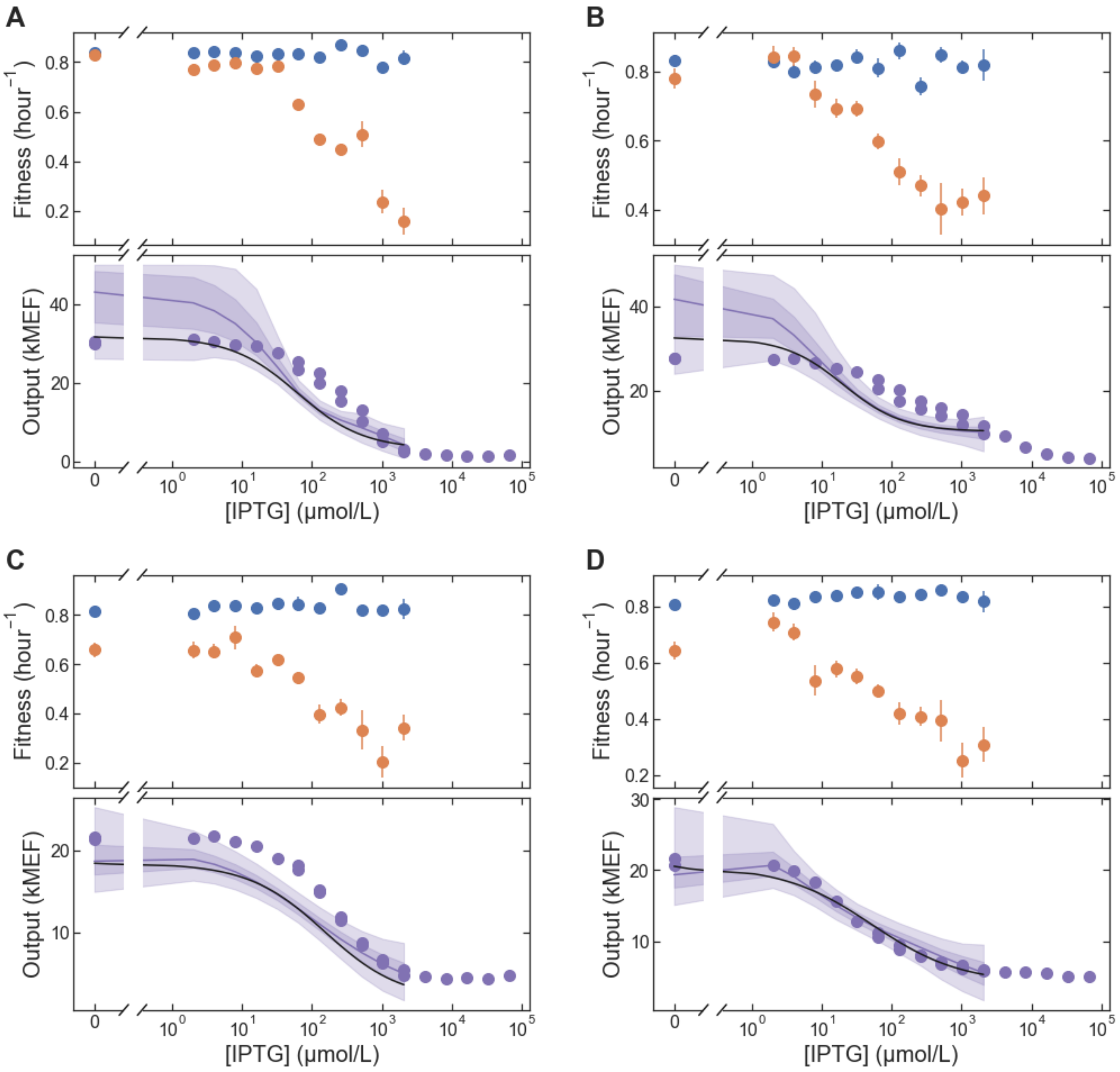

Appendix Figure S6 - Example data for fitness and dose-response curves for inverted phenotype LacI variants.

Each pair of plots shows the fitness (top) and dose-response curve (bottom) for a different LacI variant. The fitness data is from the library-scale landscape measurement. The fitness without tetracycline is shown as blue points and the fitness with tetracycline is shown as orange points. In the dose-response plots, the black lines show the results from the library-scale measurement using the Bayesian Hill equation model. The purple lines and shaded regions show the results from the library-scale measurement using the Bayesian Gaussian process (GP) model, where the line is the median GP results and the shaded regions indicate 50% and 90% credible intervals. The plotted purple points show the dose-response curves from the flow cytometry verification measurements. Error bars indicate ± one standard deviation estimated from the least-squares fit (fitness, top plots) or the Bayesian posterior (output, bottom plots), and are often within the markers.

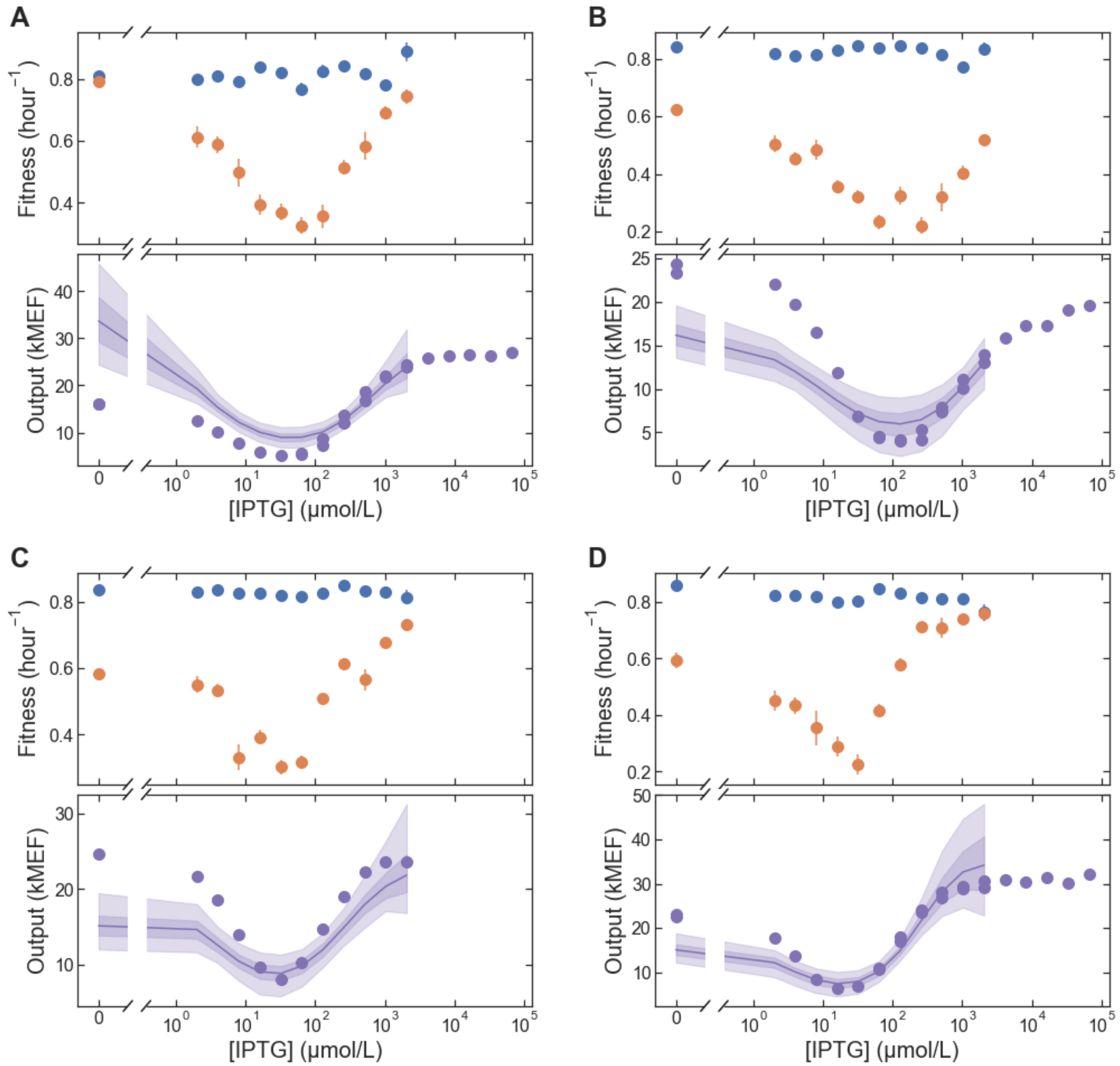

Appendix Figure S7 - Example data for fitness and dose-response curves for band-stop phenotype LacI variants.

Each pair of plots shows the fitness (top) and dose-response curve (bottom) for a different LacI variant. The fitness data is from the library-scale landscape measurement. The fitness without tetracycline is shown as blue points and the fitness with tetracycline is shown as orange points. In the dose-response plots, the black lines show the results from the library-scale measurement using the Bayesian Hill equation model. The purple lines and shaded regions show the results from the library-scale measurement using the Bayesian Gaussian process (GP) model, where the line is the median GP results and the shaded regions indicate 50% and 90% credible intervals. The plotted purple points show the dose-response curves from the flow cytometry verification measurements. Error bars indicate ± one standard deviation estimated from the least-squares fit (fitness, top plots) or the Bayesian posterior (output, bottom plots), and are often within the markers.

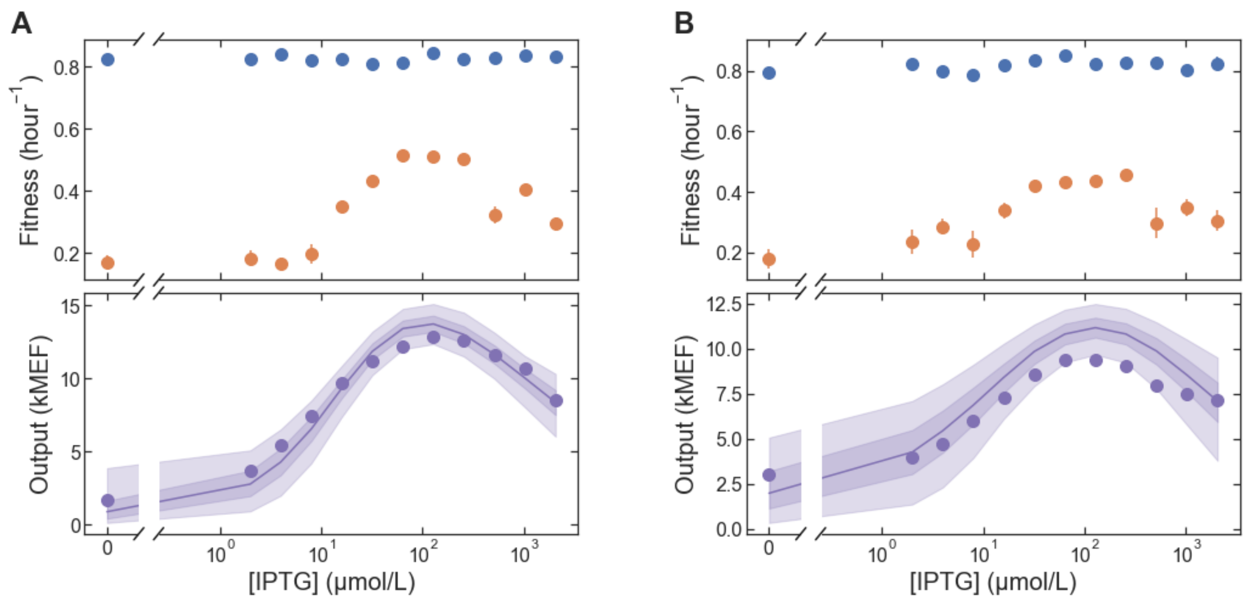

Appendix Figure S8 - Example data for fitness and dose-response curves for band-pass phenotype LacI variants.

Each pair of plots shows the fitness (top) and dose-response curve (bottom) for a different LacI variant. The fitness data is from the library-scale landscape measurement. The fitness without tetracycline is shown as blue points and the fitness with tetracycline is shown as orange points. In the dose-response plots, the black lines show the results from the library-scale measurement using the Bayesian Hill equation model. The purple lines and shaded regions show the results from the library-scale measurement using the Bayesian Gaussian process (GP) model, where the line is the median GP results and the shaded regions indicate 50% and 90% credible intervals. The plotted purple points show the dose-response curves from the flow cytometry verification measurements. Error bars indicate ± one standard deviation estimated from the least-squares fit (fitness, top plots) or the Bayesian posterior (output, bottom plots), and are often within the markers.

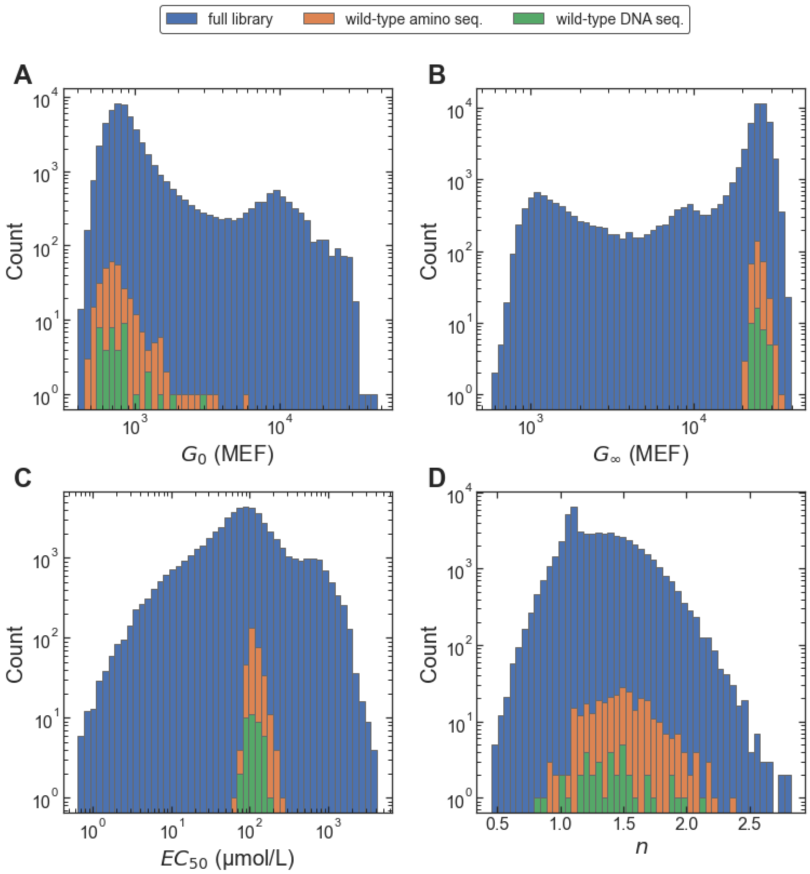

Appendix Figure S9 - Distributions of Hill equation parameters measured for the full library and the wild-type variants.

In each plot, the distribution from the full LacI library is shown in blue, the distribution from variants with the wild-type LacI DNA coding sequence is shown in green, and the distribution from variants with synonymous mutations (i.e. wild-type amino acid sequence but non-wild-type DNA sequence) is shown in orange. The green and orange data are only shown for variants with zero unintended mutations in the plasmid (i.e. no mutations in regions of the plasmid other than the *lacI* coding DNA sequence). The Kolmogorov-Smirnov test was used to compare the distributions of Hill equation parameters between variants with the wild-type DNA sequence (green, 39 variants) and synonymous mutations (orange, 310 variants). The resulting p-values (0.71, 0.40, 0.28, and 0.17 for G_0, G_∞, EC_{50}, and n respectively, Kolmogorov-Smirnov test) indicate that there were no significant differences between them.

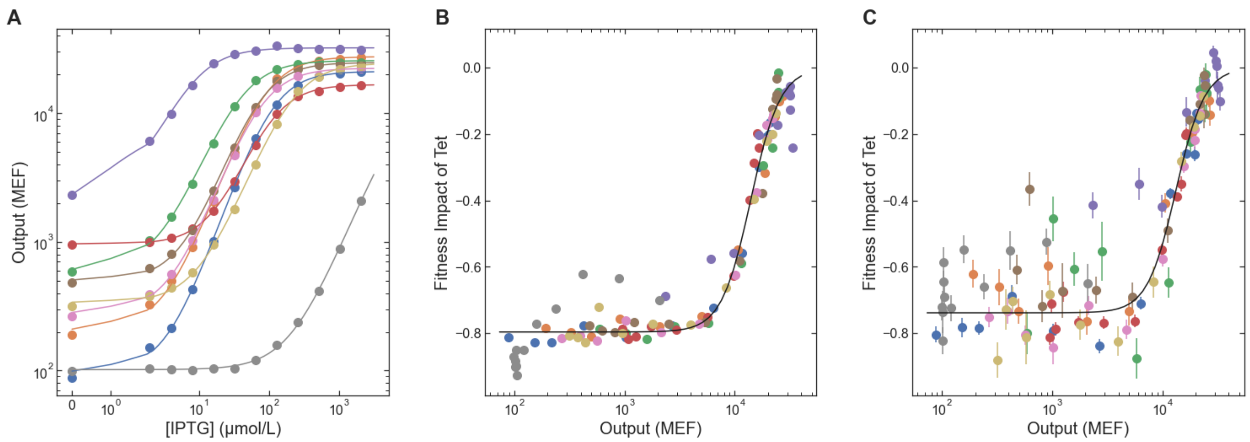

Appendix Figure S10 - Calibration data for the determination of dose-response curves using barcode sequencing.

A Dose-response curves measured with flow cytometry for nine LacI variants used to calibrate library-scale measurement.

B-C The fitness impact of tetracycline (from the library-scale measurement) plotted vs. gene expression output (from flow cytometry of individual variants). The fitness impact of tetracycline is defined as the decrease in fitness (*E. coli* growth rate) measured with tetracycline vs. without tetracycline normalized by the fitness measured without tetracycline, i.e. ($\mu^{tet}/\mu^{0} - 1$) from Eq. (9) in the Materials and Methods. (B) Data from a small-scale test library containing only the calibration variants. (C) Data from measurement of the full library. Results for different calibration variants are shown with different colored symbols. The solid black lines in show the results of a fit using Eq. (9). Error bars indicate ± one standard deviation estimated from the least-squares fit. The results for the full library measurement are noisier than the small-scale test because of the lower read count per variant (approximately 100-fold difference), but the general trend and the fits for both results are similar.

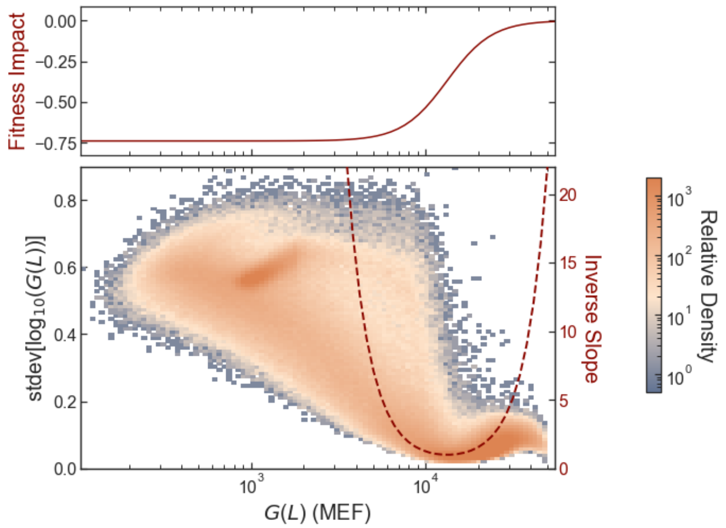

Appendix Figure S11 - Gene expression measurement uncertainty for library-scale measurement of LacI dose-response curves.

The bottom plot shows the uncertainty vs. the median for the gene expression values, $G(L)$, estimated using the Gaussian process (GP) inference model (Materials and Methods). Here, the uncertainty is the posterior standard deviation of $\log_{10}(G(L))$ from the Bayesian inference. The color map shows the relative density of data for all of the gene expression levels from the library-scale measurements (i.e. for every variant at every IPTG concentration). The uncertainty is relatively high for $G(L) < 10^4$ because the fitness impact of tetracycline used for the measurement (top plot, taken from Appendix Figure S10c) is approximately constant over that range. To illustrate this further, the dark red dashed line in the bottom plot shows the inverse slope of the fitness impact curve. For $G(L)$ between about 7,000 MEF and 25,000 MEF, the uncertainty is approximately proportional to the inverse slope, as expected. Outside that range, the uncertainly is no longer proportional to the inverse slope of the fitness impact curve because the posterior $G(L)$ estimate is constrained by the prior used in the Bayesian inference to the range 100 MEF < $G(L)$ < 50,000 MEF.

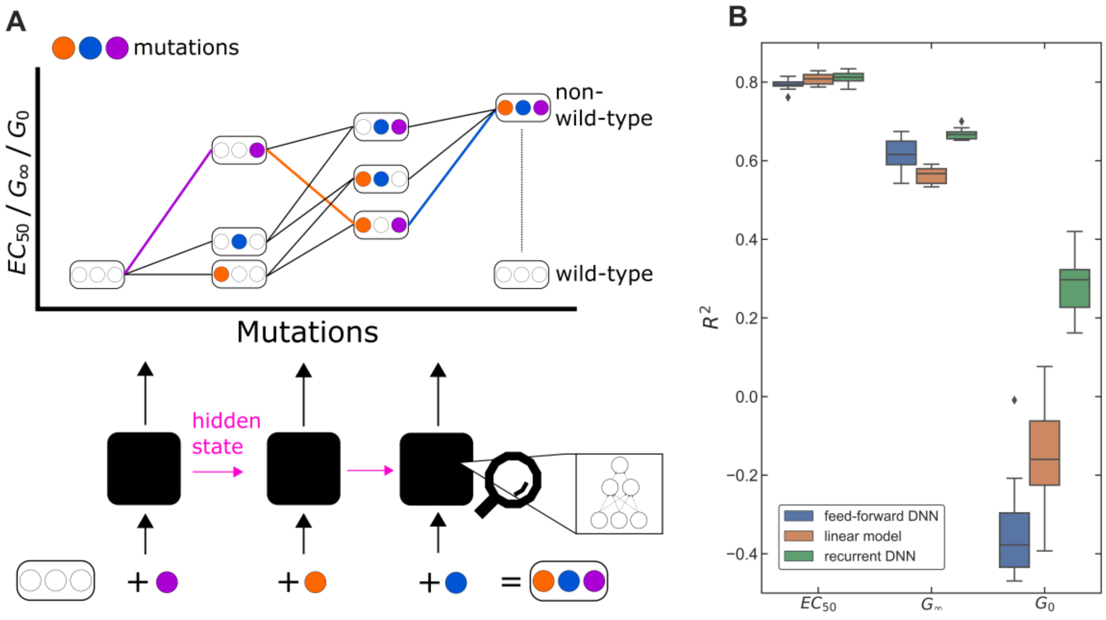

Appendix Figure S12 - Deep neural network (DNN) model.

A Schematic illustration of recurrent deep neural network model architecture. Using the wild-type LacI amino acid sequence as a starting point, the model predicts the Hill equation parameters for non-wild-type sequences by composing the individual parameter changes due to sequential amino acid substitutions along a mutational path. These changes are then added together to predict the final parameter values. All paths to a non-wild-type sequence converge to the same value, and this fact is leveraged to build a recurrent neural network model that learns to predict the effects of each substitution, given all previous substitutions. Potential non-additive effects are captured by the hidden state of the model, which predicts the change in parameter value for the most recent substitution and serves as a set of latent variables for predicting subsequent substitutions. Note that variants with intermediate sequences may be present in the library, but this is not necessary to train the model. The model will still learn to predict intermediate steps in the path, even if that data is not present.

B Performance of recurrent DNN model compared with linear-additive model and feed-forward DNN model. The boxplot summarizes the R^2 values for ten cross-validation tests for each model. The recurrent DNN model (green) outperforms both the linear-additive (orange) and feed-forward (blue) models, giving the highest R^2 values for the Hill equation parameters G_0 and G_∞. For EC_{50}, the recurrent DNN model and the linear-additive model give similar R^2 values.

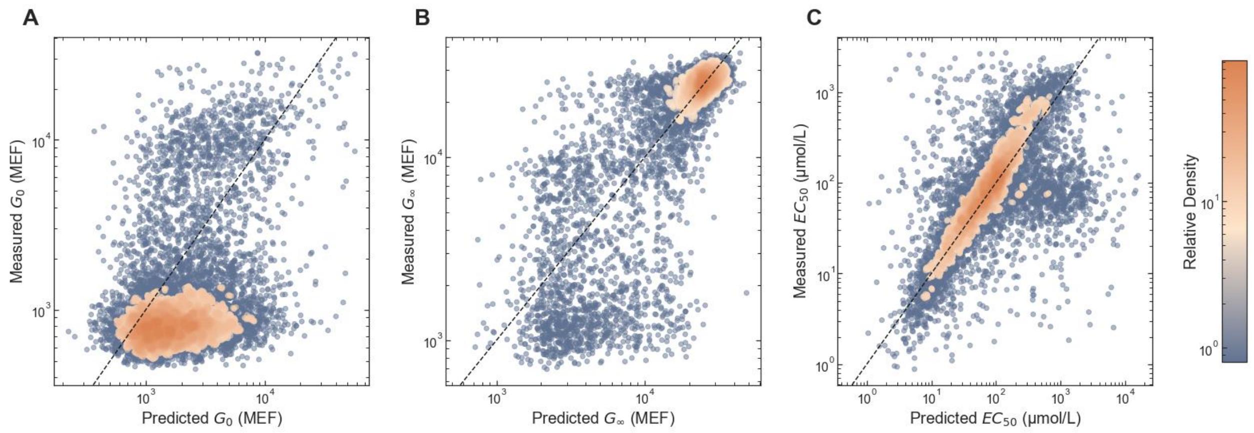

Appendix Figure S13 - Prediction of LacI variant phenotypes with recurrent deep neural network (DNN) model.

Measured vs. predicted values for each Hill equation parameter. The plotted results only show holdout data not used in model training. Predictions are taken from the variational posterior mean for each Hill equation parameter. Measured values are the posterior medians from the Bayesian fits to the experimental data.

A-B G_0 and G_∞ are given in molecules of equivalent fluorophore (MEF) based on the calibration with flow cytometry data (see Materials and Methods).

C The cluster of points with measured EC_{50} near 100 µmol/L and predicted EC_{50} near 1000 µmol/L correspond to variants for which the DNN model provided better EC_{50} and G_∞ estimates than the measurement, because EC_{50} was near or above the maximum ligand concentration used. For these points, the nearly constant dose-response across the measured concentration range resulted in a large uncertainty for EC_{50} and a posterior median near the median of the EC_{50} prior (100 µmol/L).

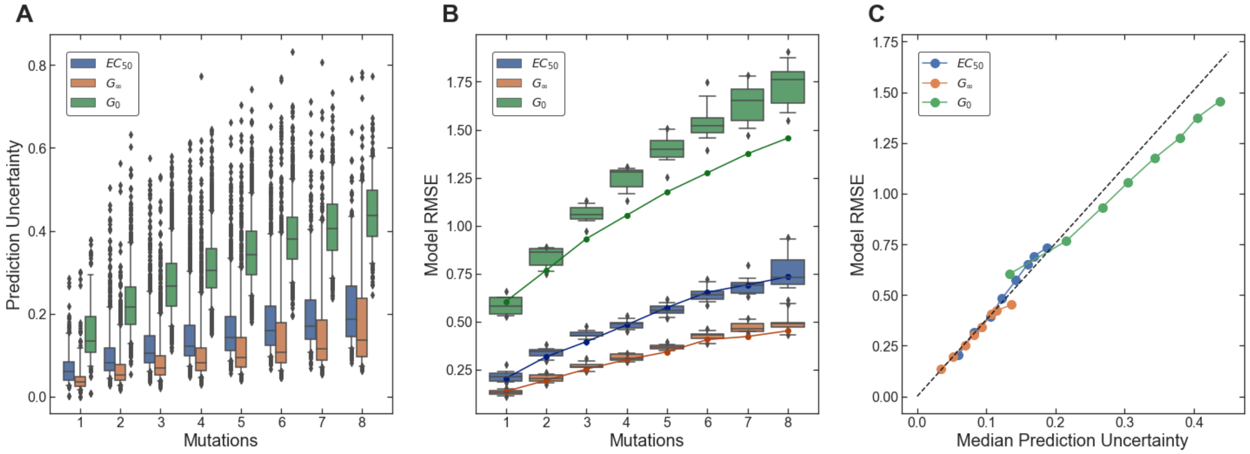

Appendix Figure S14 DNN model prediction uncertainty and root-mean-square error (RMSE).

A Model prediction uncertainty for the base-ten logarithm of each Hill equation parameter as a function of the number of amino acid substitutions relative to the wild-type LacI sequence. The boxplot shows the distribution of posterior standard deviation values for the set of variants with the indicated number of substitutions.

B Model RMSE for each Hill equation parameter as a function of the number of substitutions relative to the wild-type sequence. RMSE is the root-mean-square difference between the model prediction and experimental measurement for the base-ten logarithm of each Hill equation parameter. The boxplot shows the distribution of RMSE values from the ten cross-validation tests for the recurrent DNN model. Solid lines with points show the RMSE for the holdout data not used for training.

Both the prediction uncertainty (A) and the RMSE (B) increase with increasing number of substitutions relative to the wild-type sequence.

C Model RMSE as a function of the median prediction uncertainty for each Hill equation parameter. Each plotted point is for a different number of substitutions. The RMSE values are from the holdout data, i.e. the solid lines with points from (**B**). The median prediction accuracy values are from the distributions plotted in (**A**). The dashed line indicates the multiplicative factor (approximately 3.8) used to correct the uncertainty values (see Materials and Methods).

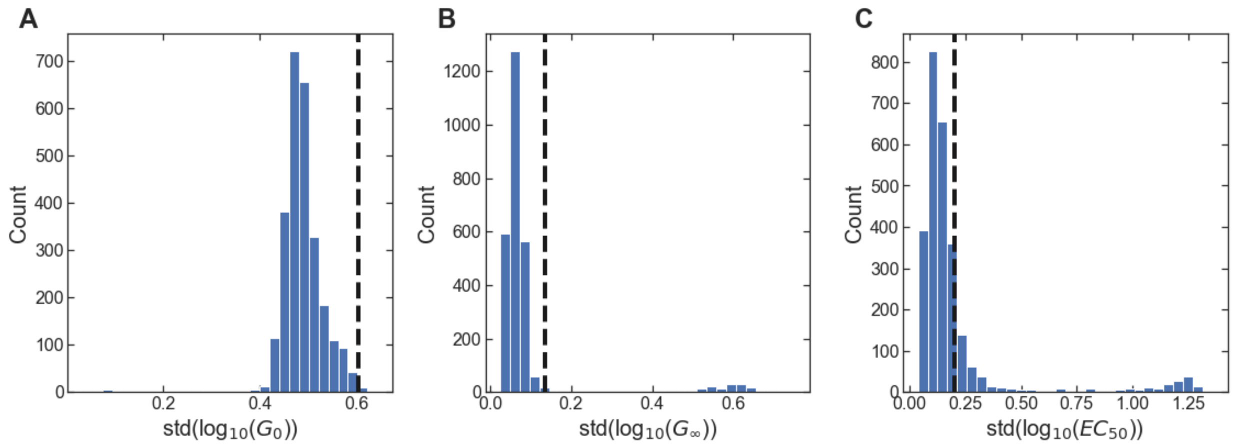

Appendix Figure S15 - Comparison between the DNN model root-mean-square error (RMSE) and the measurement uncertainty for single mutants.

The histograms show distributions of the measurement uncertainty for LacI variants with single amino acid substitutions (relative to the wild type) for each Hill equation parameter. For comparison, the dashed line in each plot shows the RMSE for the DNN model predictions for single-substitution hold-out data. The model RMSE as a function of the number of substitutions is shown in Appendix Figure S14B.

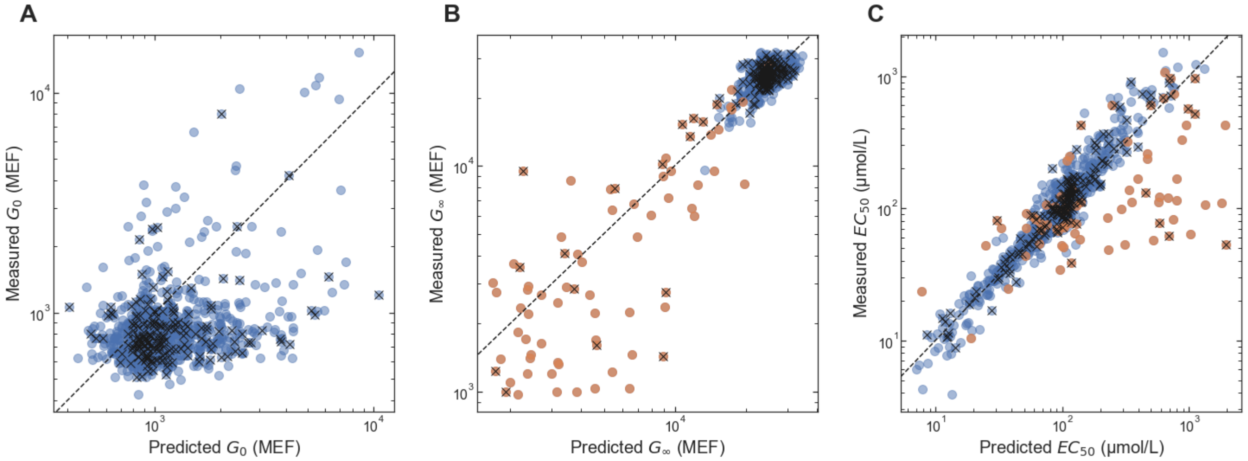

Appendix Figure S16 - Measured vs. predicted values from DNN model for single amino acid substitutions.

The plots compare the DNN model predictions (x-axis) with the measured values (y-axis) for the Hill equation parameters for LacI variants with single amino acid substitutions. Blue symbols show data for all of the single-substitution variants in the library. Orange symbols show data for variants with a high uncertainty for the measured EC_{50} (std($\log_{10}$(EC_{50})) > 0.35). The EC_{50} uncertainty for those variants was relatively high either because G_∞ was similar to G_0 and/or because EC_{50} was near or above the maximum ligand concentration used (2048 µmol/L). For those variants, in the analysis of single-mutant effects, the DNN model result for G_∞ and EC_{50} was used in place of the experimental result. Points marked with an 'x' were in the holdout data not used for model training. In all plots, G_0 and G_∞ are given in molecules of equivalent fluorophore (MEF).

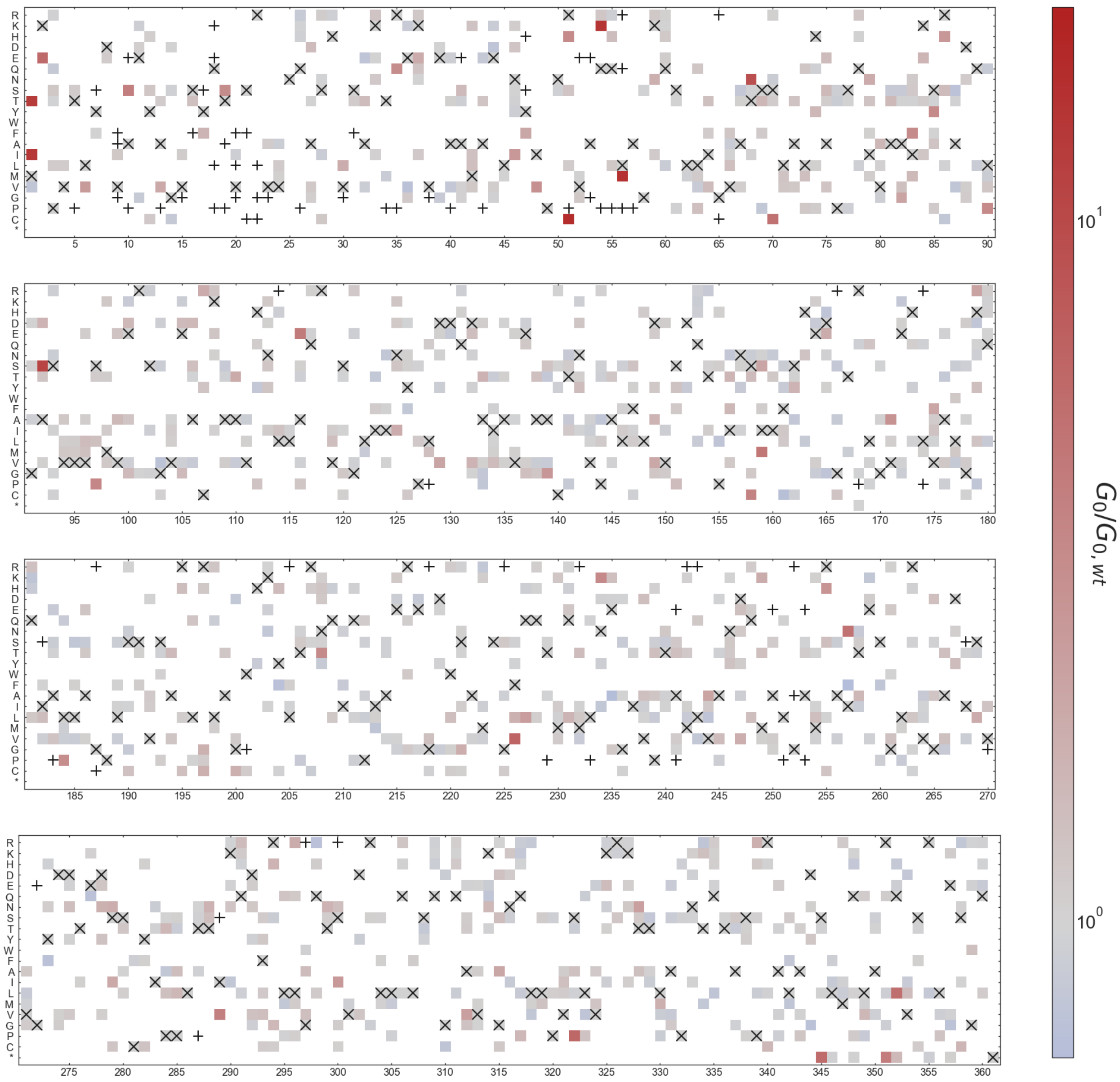

Appendix Figure S17 - Heat map of single substitution effects on G_0.

The heat map indicates the change in G_0 for all amino acid substitutions measured as single substitutions in the LacI library. The amino acid residue number is plotted as the x-axis and the possible amino acid substitutions as the y-axis. The wild-type amino acid at each residue is marked with an 'X'. The 83 substitutions that are completely missing from the landscape dataset and that have been shown by previous work to result in constitutively high $G(L)$[1,2] are each marked with a '+'. The data plotted in this figure is included in Dataset EV1.

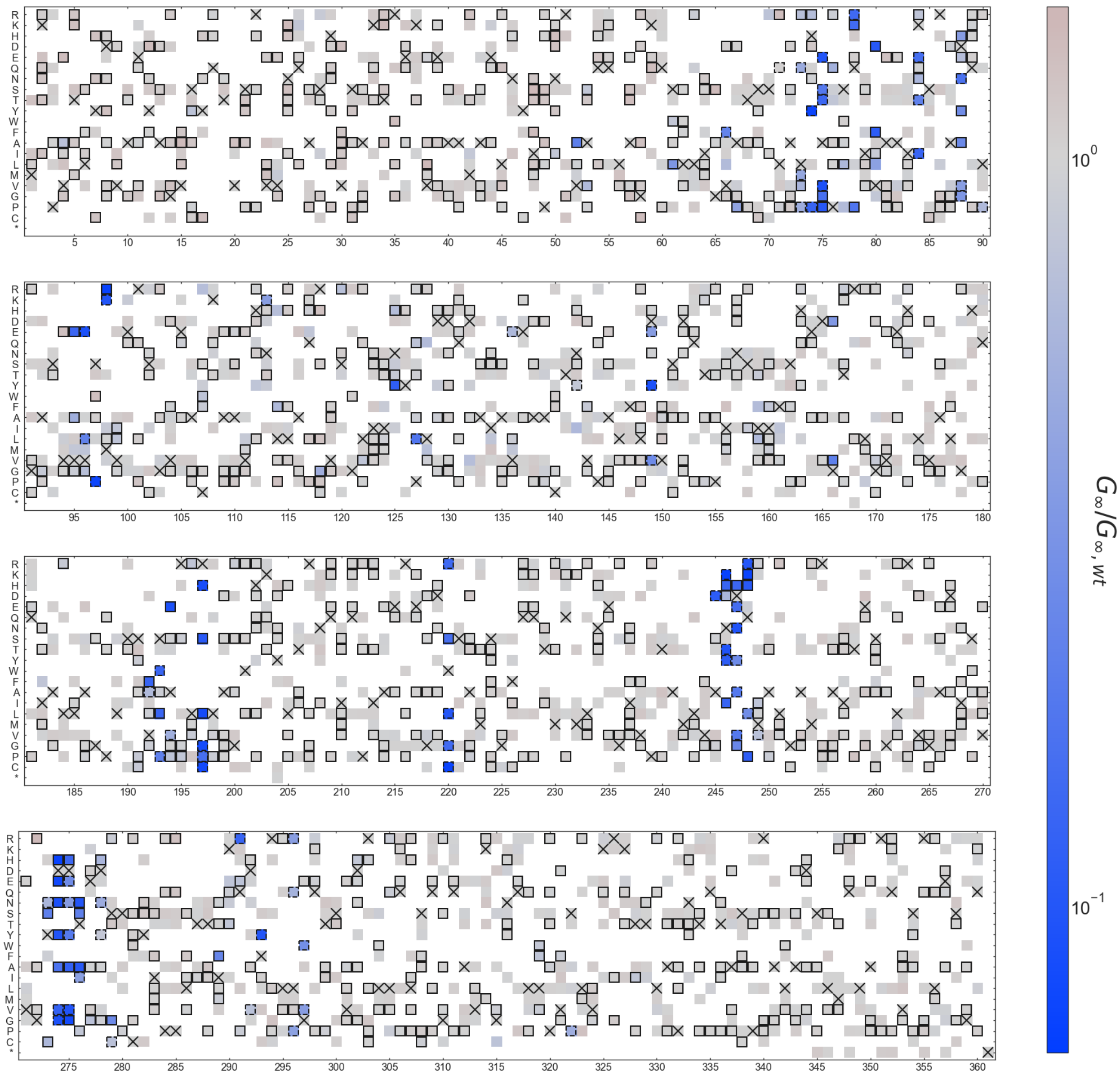

Appendix Figure S18 - Heat map of single substitution effects on *G*∞.

The heat map indicates the change in G_∞ for all amino acid substitutions measured as single substitutions in the LacI library and/or predicted from the DNN model. The amino acid residue number is plotted as the x-axis and the possible amino acid substitutions as the y-axis. The wild-type amino acid at each residue is marked with an 'X'. Substitutions with effects that are predicted from the DNN model are outlined with solid lines. Substitutions with high measurement uncertainty, where the DNN model result was used in place of the experimental result are outlined with dashed lines (see Appendix Figure S16). The data plotted in this figure is included in Dataset EV1.

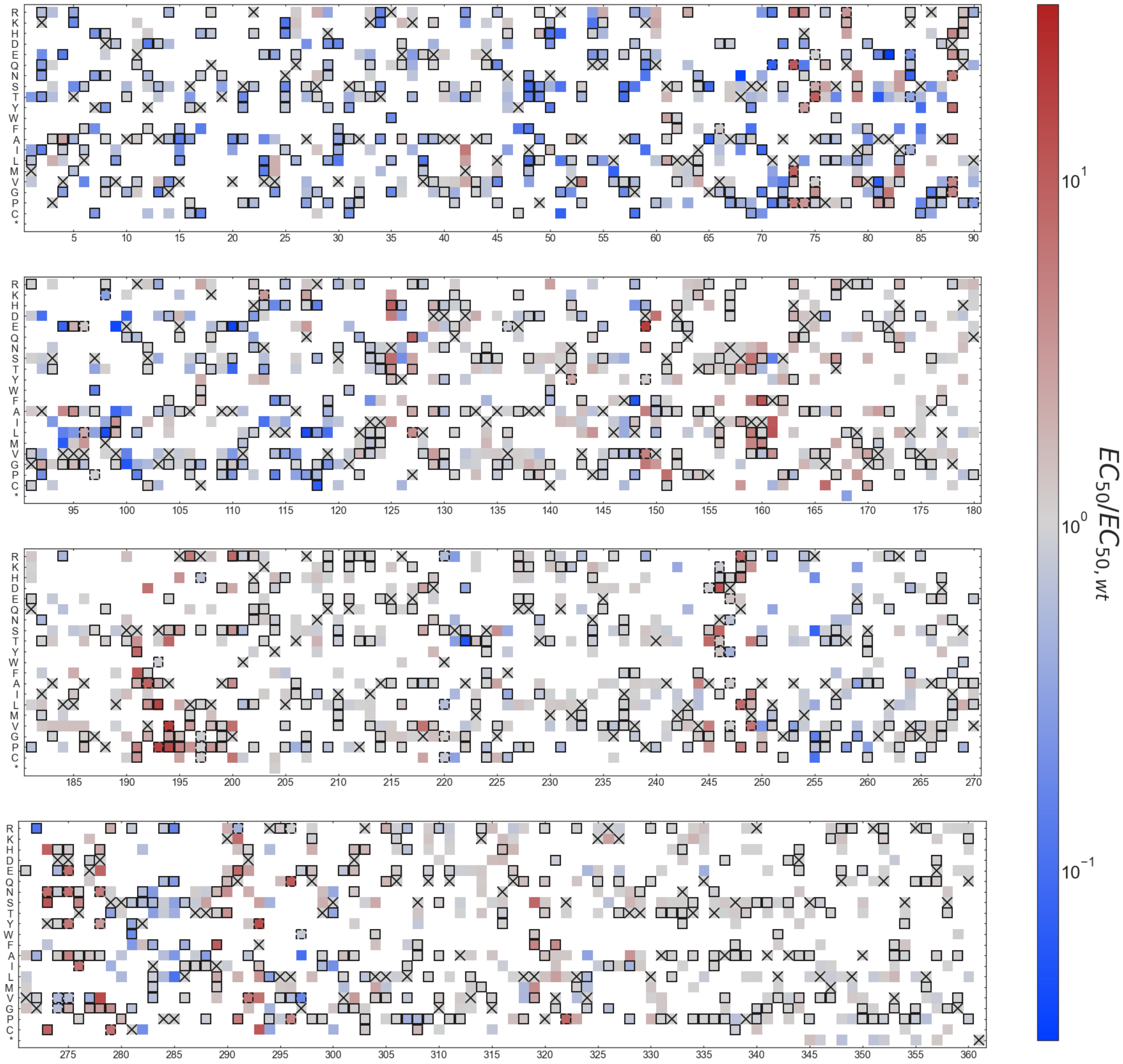

Appendix Figure S19 - Heat map of single substitution effects on EC_{50}.

The heat map indicates the change in EC_{50} for all amino acid substitutions measured as single substitutions in the LacI library and/or predicted from the DNN model. The amino acid residue number is plotted as the x-axis and the possible amino acid substitutions as the y-axis. The wild-type amino acid at each residue is marked with an ‘X’. Substitutions with effects that are predicted from the DNN model are outlined with solid lines. Substitutions with high measurement uncertainty, where the DNN model result was used in place of the experimental result are outlined with dashed lines (see Appendix Figure S16). The data plotted in this figure is included in Dataset EV1.

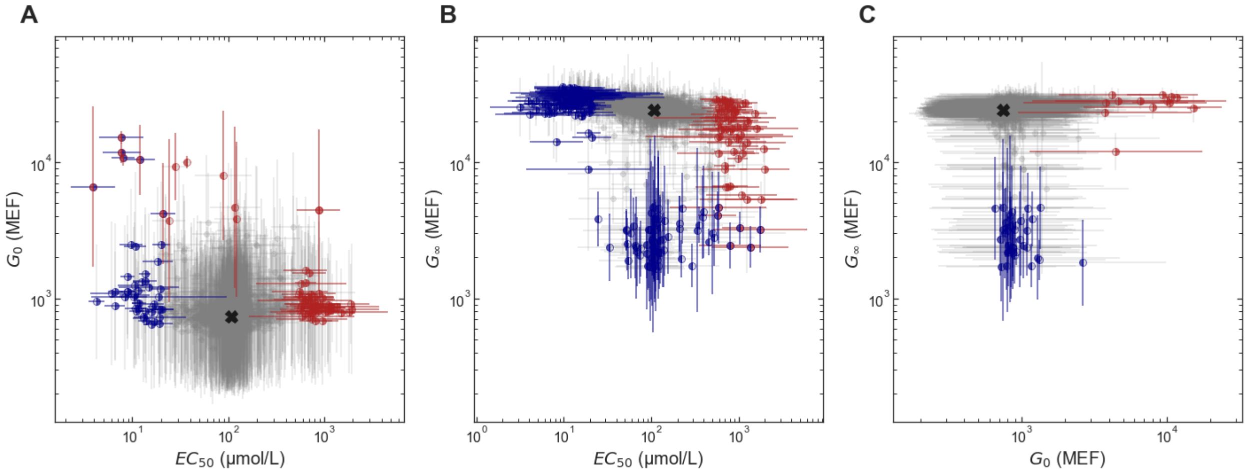

Appendix Figure S20 - Multiparametric impact of amino acid substitutions on allosteric function of LacI.

Each plot shows the joint effect of single amino acid substitutions on two Hill equation parameters. In each plot, substitutions that change both Hill equation parameters by less than 5-fold are shown as light gray points, and substitutions that change one or both Hill equation parameters by more than 5-fold are shown as red or blue points with error bars. As in Fig. 3 (in the main manuscript), red indicates a decrease in Hill equation parameter and blue indicates an increase. The left half of each symbol and the y-error bar are colored based on the y-axis parameter; the right half of each symbol and the x-error bars are colored based on the x-axis parameter. The wild-type phenotype is indicated with a black 'X' in each plot. In all plots, G_0 and G_∞ are given in molecules of equivalent fluorophore (MEF). Error bars indicate ± one standard deviation estimated from the Bayesian posterior.

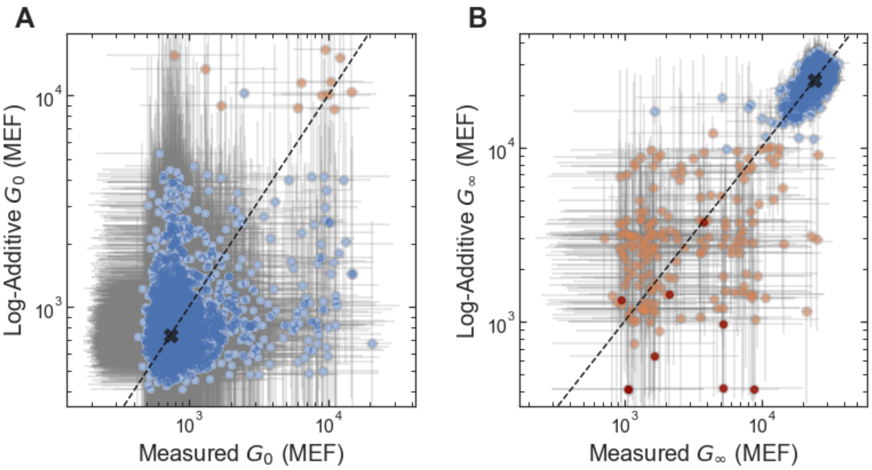

Appendix Figure S21 - Comparison between log-additive prediction and measurement for double-substitution variants for G_0 and G_∞.

The log-additive G_0 and G_∞ for double-substitution LacI variants (i.e. two amino acid substitutions) was calculated assuming log-additivity of the effect of each single substitution relative to the wild-type, e.g. $\log(G_{\infty,AB}/G_{\infty,wt}) = \log(G_{\infty,A}/G_{\infty,wt}) + \log(G_{\infty,B}/G_{\infty,wt})$, where '*wt*' indicates the wild-type, '*A*' and '*B*' indicate the single-substitution variants, and '*AB*' indicates the double-substitution variant. Similar to the analysis of genotype-phenotype landscape data for green fluorescent protein, the log-additive predictions were constrained to within the minimum and maximum measured G_0 and G_∞ values: 410 MEF < $G_{0,AB}$, $G_{\infty,AB}$ < 39,000 MEF.

A Orange points mark double-substitution variants in which one of the single substitutions causes a greater than 10-fold change in G_0.

B Orange points mark double-substitution variants in which one of the single substitutions causes a greater than 2.5-fold change in G_∞, and dark red points mark double-substitution variants in which both single substitutions cause a greater than 2.5-fold change in G_∞. For G_∞, data were only used for variants with EC_{50} < 500 µmol/L to ensure that the $G(L)$ level was near saturation at the highest IPTG concentration used (2048 µmol/L).

In each plot, the wild-type parameter value is marked with a black 'X'. For this analysis, only experimental data was used (no results from the DNN model). Error bars for the measured result indicate ± one standard deviation estimated from the Bayesian posterior; error bars for the lag-additive result indicate ± one standard deviation propagated from the Bayesian posterior uncertainties of the single-substitution results.

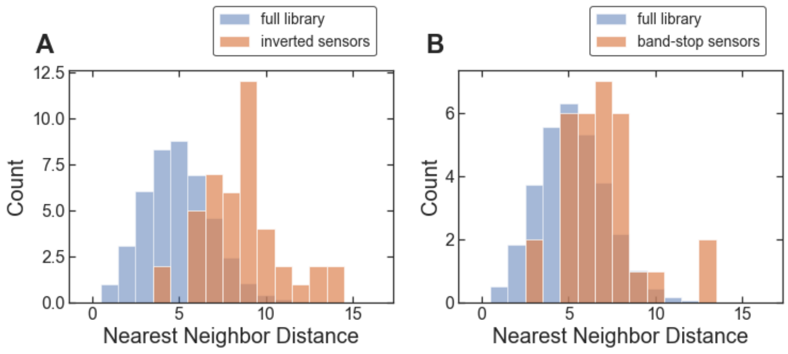

Appendix Figure S22 Nearest neighbor distance histograms for inverted and band-stop LacI variants.

In each plot, the orange bars show the distribution of nearest neighbor Hamming distance for the amino acid sequences for strongly inverted (A) and strong band-stop (B) variants, and the blue bars show the distribution of nearest neighbor Hamming distance for a similar number of randomly selected sequences from the full library. The full-library histograms (blue bars) are averaged over 1000 iterations of randomly selected sequences.

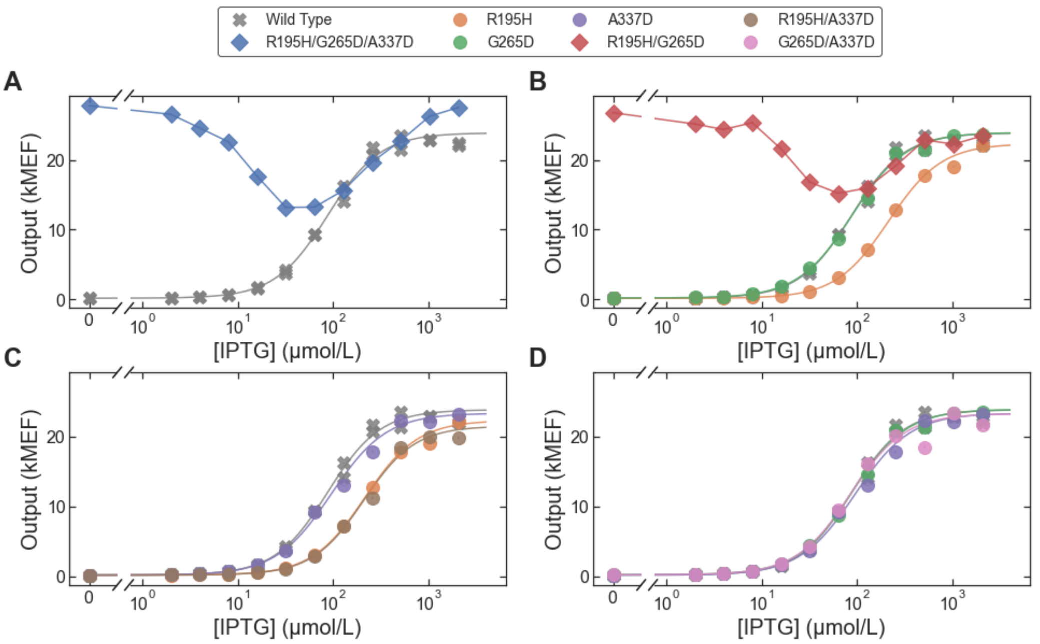

Appendix Figure S23 - The band-stop phenotype emerges from the combination of two amino acid substitutions.

A Dose-response curves measured with flow cytometry for wild-type LacI and a strong band-stop variant identified from the library with only three amino acid substitutions (R195H, G265D, A337D).

B-D Dose-response curves for LacI variants containing single- and double-substitution permutations of the three substitutions in the band-stop variant. Each plot shows two LacI variants containing single substitutions and one LacI variant containing both substitutions. The single substitutions R195H (orange) or G265D (green) result in sigmoidal dose-response curves similar to the wild-type, but the combination of the two, R195H/G265D (red), results in a band-stop phenotype (**B**). All other combinations of single- and double-substitutions result in sigmoidal dose-response curves similar to the wild-type (**C-D**).

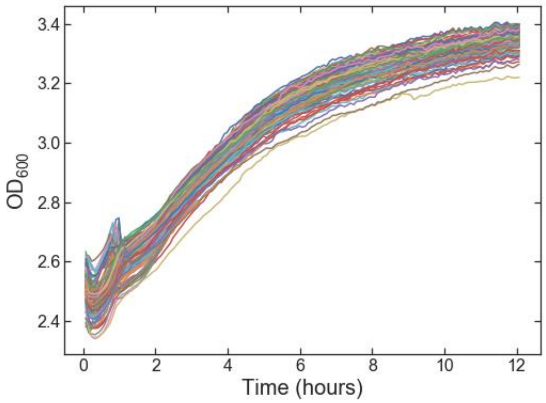

Appendix Figure S24 - Measurement of OD_{600} vs. time for *E. coli* growth during initial, 12-hour incubation in automated culture protocol.

Measurements are shown for all 96 wells in the growth plate during the library-scale measurement of LacI dose-response curves. The OD_{600} measurement is not corrected for the zero-OD offset caused by the membrane (approximately 1.7).

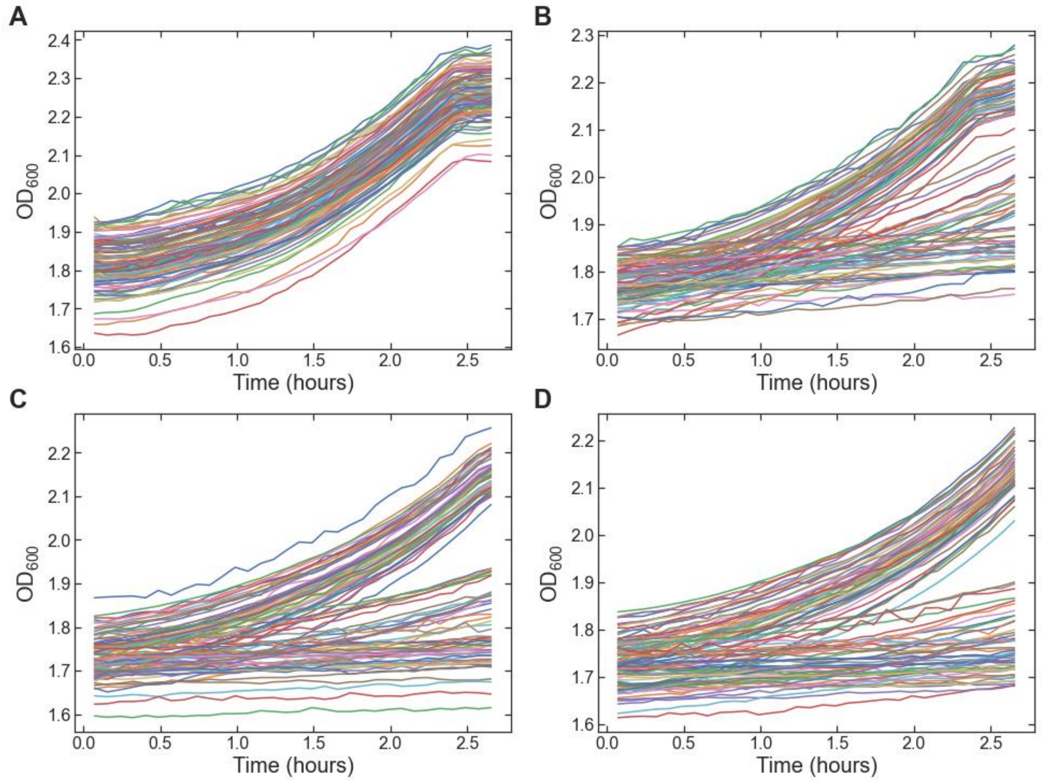

Appendix Figure S25 - Measurement of OD_{600} vs. time for *E. coli* in Growth Plates 1-4.

Measurements are shown for all 96 wells in each growth plate during the library-scale measurement of LacI dose-response curves.

A Growth Plate 1.

B Growth Plate 2.

C Growth Plate 3.

D Growth Plate 4.

The OD_{600} measurements are not corrected for the zero-OD offset caused by the membrane (approximately 1.7). Note that the flattening of the growth curves at time ≈ 2.4 hours in (A) and (B), is not the onset of stationary phase. Instead, it is an indication of a diauxic shift which was used as a marker for the maximum cell density at which the cells remained in exponential growth phase. An example set of full growth curves is shown in Appendix Figure S26.

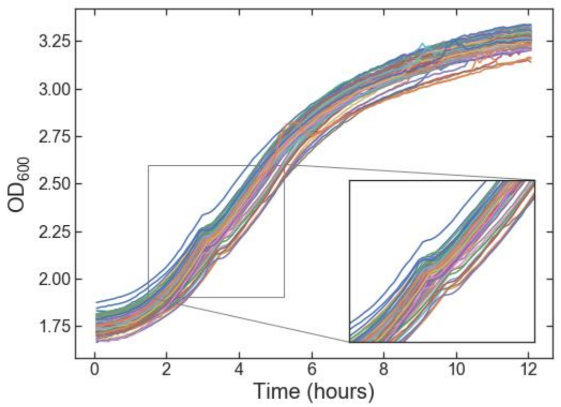

Appendix Figure S26 - Example of OD_{600} vs. time measurement for a full *E. coli* growth curve.

The inset shows a zoomed-in view of the short flat portion of the growth curves indicating the onset of a diauxic shift. This diauxic shift was used as a marker for the maximum cell density at which cells remained in exponential growth.

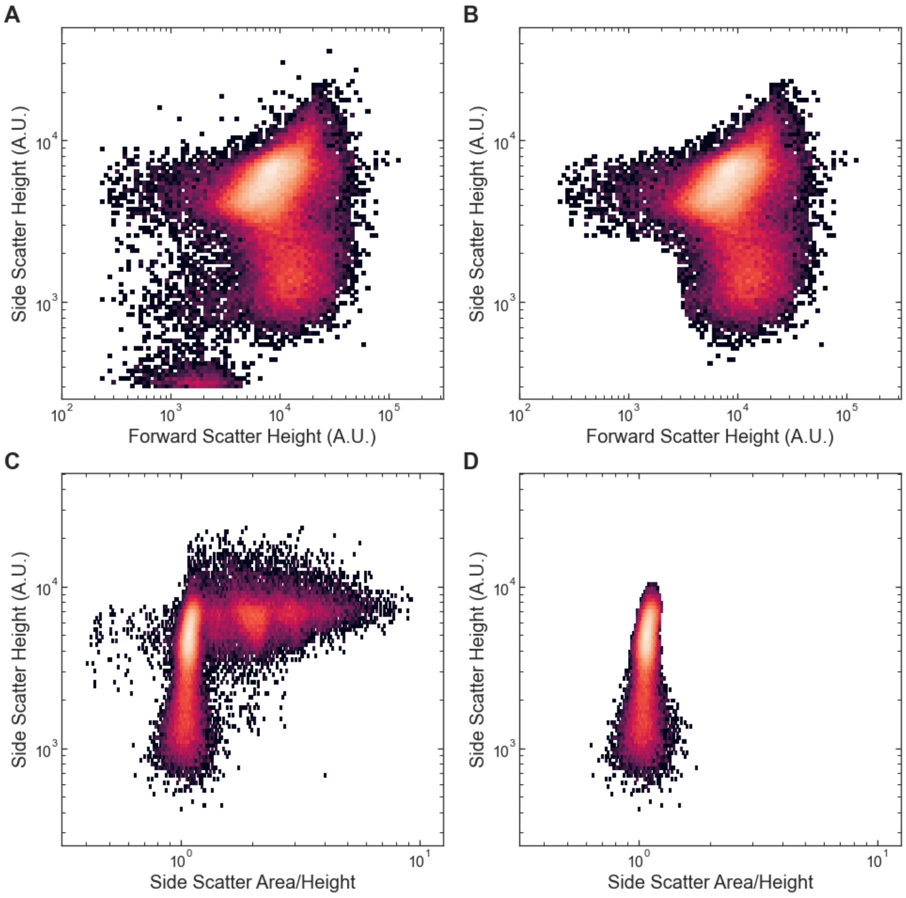

Appendix Figure S27 - Flow cytometry gating example.

A Side scatter vs. forward scatter plot before automated cell gating, showing both cell and non-cell detection events.

B Side scatter vs. forward scatter plot after automated cell gating, showing only events most likely to be cell events.

C Side scatter vs. side scatter area/height plot before automated singlet gating, showing both singlet and multiplet cell detection events.

D Side scatter vs. side scatter area/height plot after automated singlet gating, showing only singlet cell detection events.

number of single nucleotide polymorphisms	number of unique DNA sequences in library	number of possible DNA sequences	fraction of possible DNA sequences in library
1	313	3.24×10^{3}	9.66×10^{-2}
2	1259	1.05×10^{7}	1.20×10^{-4}
3	3023	3.39×10^{10}	8.91×10^{-8}
4	5653	1.10×10^{14}	5.16×10^{-11}
5	8058	3.54×10^{17}	2.28×10^{-14}
6	9440	1.14×10^{21}	8.27×10^{-18}
7	9451	3.68×10^{24}	2.57×10^{-21}
8	8317	1.18×10^{28}	7.03×10^{-25}
9	6187	3.81×10^{31}	1.63×10^{-28}
10	4266	1.22×10^{35}	3.49×10^{-32}
11	2689	3.92×10^{38}	6.85×10^{-36}
12	1507	1.26×10^{42}	1.20×10^{-39}
13	773	4.03×10^{45}	1.92×10^{-43}
14	385	1.29×10^{49}	2.98×10^{-47}
15	194	4.13×10^{52}	4.70×10^{-51}
total	62470	1.95×10^{515}	3.20×10^{-511}

Appendix Table S1 - Number of measured genotypes in library for different mutational distances from wild-type *lacI*.

A characterization of the diversity found within the library in terms of in terms of single nucleotide polymorphisms of the wildtype *lacI* sequence.

domain/feature	amino acid residues
DNA binding domain	1-62
N terminal core domain	63-161, 293-318
C terminal core domain	164-290, 322-324
tetramer helix (helix 14)	340-357
within 7 Å of ligand	68-70, 73-76, 79, 125-127, 148-150, 160-161, 188, 191, 193-194, 197, 220, 245-249, 273-274, 276, 291, 293, 296
dimer interface	70-100, 221-226, 250-260, 275-285
core pivot region	150-153, 161-164, 190-193, 290-293, 318-322
helix 1	6-12
helix 2	17-25
helix 3	32-45
helix 4	50-58
β-strand A	63-68
helix 5	74-90
β-strand B	92-98
helix 6	104-116
β-strand C	121-124
helix 7	131-137
β-strand D	145-149
β-strand E	158-161
helix 8	164-175
β-strand F	182-185
helix 9	192-205
β-strand G	214-217
helix 10	222-233
β-strand H	240-244
helix 11	247-259
β-strand I	269-274
helix 12	279-281
β-strand J	287-290
helix 13	293-309
β-strand K	316-318
β-strand L	322-324

Appendix Table S2 - LacI protein structural domains and features referred to in the manuscript.

Residues within 7 Å of the ligand were determined using the crystal structure of LacI bound to IPTG (PDB ID: 1LBH). The dimer interface was taken as described by Lewis et al [3] plus residues 117 and 247-249, which are also along the dimer interface based on the published crystal structures (PDB IDs: 1LBG and 1LBH). The core pivot region was taken as described by Swint-Kruse et al.[4]. All other domains and features were taken as described by Lewis et al.[3]

substitution or domain/feature	substitution count for all variants in library (52,321 total)	percentage of all variants with substitution	substitution count for strongly inverted variants (43 total)	percentage of strongly inverted variants with substitution	p-value
D88Y	199	0.38	5	11.63	6.48×10^{-7}
V96E	139	0.27	3	6.98	2.09×10^{-4}
K84N	150	0.29	3	6.98	2.62×10^{-4}
S70I	35	0.07	2	4.65	3.86×10^{-4}
Q248H	177	0.34	3	6.98	4.25×10^{-4}
Y273H	89	0.17	2	4.65	2.47×10^{-3}
A343G	95	0.18	2	4.65	2.81×10^{-3}
V192A	98	0.19	2	4.65	2.98×10^{-3}
A135T	352	0.67	3	6.98	3.05×10^{-3}
G200S	114	0.22	2	4.65	4.01×10^{-3}
dimer interface	28175	53.85	39	90.7	2.05×10^{-7}
within 7 Å of ligand	16345	31.24	29	67.44	1.15×10^{-6}
helix 5	9766	18.67	20	46.51	2.87×10^{-5}
helix 11	5846	11.17	12	27.91	2.06×10^{-3}
strand I	2306	4.41	6	13.95	1.10×10^{-2}

Appendix Table S3 - Amino acid substitutions and structural domains or features associated with the inverted LacI phenotype.

The top portion of the table lists the amino acid substitutions that occur more frequently in the set of strongly inverted LacI variants than in the full library, with a p-value cutoff of 5×10^{-3}. The bottom portion of the table lists the structural domains or features where substitutions were found at higher frequency in the set of strongly inverted LacI variants than in the full library, with a p-value cutoff of 2.5×10^{-2}. The criteria used to select the set of strongly inverted variants and the hypergeometric test used to calculate p-values are described in the Materials and Methods section.

substitution or domain/feature	substitution count for all variants in library (52,321 total)	percentage of all variants with substitution	substitution count for strong band-stop variants (31 total)	percentage of strong band-stop variants with substitution	p-value
H179Q	35	0.07	2	6.45	2.00×10^{-4}
D292G	49	0.09	2	6.45	3.93×10^{-4}
R195H	280	0.54	3	9.68	6.10×10^{-4}
G265D	101	0.19	2	6.45	1.65×10^{-3}
V4A	117	0.22	2	6.45	2.21×10^{-3}
G178D	144	0.28	2	6.45	3.32×10^{-3}
R351G	154	0.29	2	6.45	3.78×10^{-3}
A92V	533	1.02	3	9.68	3.82×10^{-3}
C terminal core domain	40858	78.09	31	100	4.67×10^{-4}
strand J	1795	3.43	4	12.9	2.08×10^{-2}
helix 9	8520	16.28	10	32.26	2.14×10^{-2}

Appendix Table S4 - Amino acid substitutions and structural domains or features associated with the band-stop LacI phenotype.

The top portion of the table lists the amino acid substitutions that occur more frequently in the set of strong band-stop LacI variants than in the full library, with a p-value cutoff of 5×10^{-3}. The bottom portion of the table lists the structural domains or features where substitutions were found at higher frequency in the set of strong band-stop LacI variants than in the full library, with a p-value cutoff of 2.5×10^{-2}. The criteria used to select the set of strong band-stop variants and the hypergeometric test used to calculate p-values are described in the Materials and Methods section.

wells	IPTG concentration (μmol/L)	tetracycline concentration (μg/mL)*	final OD_{600} Growth Plate 1	final OD_{600} Growth Plate 2	final OD_{600} Growth Plate 3	final OD_{600} Growth Plate 4
A1, C1, E1, G1	0	0	0.525 ± 0.017	0.532 ± 0.017	0.522 ± 0.036	0.472 ± 0.010
B1, D1, F1, H1	0	20	0.519 ± 0.012	0.151 ± 0.054	0.012 ± 0.007	0.034 ± 0.029
A2, C2, E2, G2	2	0	0.542 ± 0.012	0.542 ± 0.016	0.508 ± 0.017	0.485 ± 0.009
B2, D2, F2, H2	2	20	0.528 ± 0.010	0.165 ± 0.027	0.038 ± 0.034	0.040 ± 0.033
A3, C3, E3, G3	4	0	0.556 ± 0.026	0.548 ± 0.012	0.511 ± 0.015	0.491 ± 0.018
B3, D3, F3, H3	4	20	0.538 ± 0.020	0.184 ± 0.024	0.056 ± 0.025	0.034 ± 0.033
A4, C4, E4, G4	8	0	0.559 ± 0.026	0.549 ± 0.021	0.518 ± 0.006	0.493 ± 0.005
B4, D4, F4, H4	8	20	0.555 ± 0.008	0.135 ± 0.051	0.036 ± 0.052	0.038 ± 0.044
A5, C5, E5, G5	16	0	0.533 ± 0.024	0.578 ± 0.015	0.519 ± 0.027	0.486 ± 0.005
B5, D5, F5, H5	16	20	0.552 ± 0.027	0.080 ± 0.017	0.031 ± 0.013	0.027 ± 0.017
A6, C6, E6, G6	32	0	0.536 ± 0.017	0.567 ± 0.027	0.514 ± 0.024	0.496 ± 0.030
B6, D6, F6, H6	32	20	0.539 ± 0.011	0.148 ± 0.028	0.065 ± 0.025	0.048 ± 0.034
A7, C7, E7, G7	64	0	0.551 ± 0.015	0.548 ± 0.022	0.532 ± 0.020	0.480 ± 0.022
B7, D7, F7, H7	64	20	0.540 ± 0.014	0.147 ± 0.010	0.049 ± 0.009	0.063 ± 0.014
A8, C8, E8, G8	128	0	0.544 ± 0.012	0.558 ± 0.020	0.512 ± 0.023	0.486 ± 0.015
B8, D8, F8, H8	128	20	0.541 ± 0.015	0.230 ± 0.030	0.117 ± 0.037	0.094 ± 0.033
A9, C9, E9, G9	256	0	0.528 ± 0.019	0.529 ± 0.020	0.500 ± 0.004	0.461 ± 0.015
B9, D9, F9, H9	256	20	0.526 ± 0.026	0.300 ± 0.029	0.192 ± 0.021	0.123 ± 0.015
A10, C10, E10, G10	512	0	0.534 ± 0.032	0.520 ± 0.025	0.493 ± 0.014	0.479 ± 0.017
B10, D10, F10, H10	512	20	0.520 ± 0.002	0.321 ± 0.032	0.217 ± 0.029	0.163 ± 0.035
A11, C11, E11, G11	1024	0	0.500 ± 0.012	0.526 ± 0.024	0.467 ± 0.024	0.467 ± 0.011
B11, D11, F11, H11	1024	20	0.492 ± 0.024	0.327 ± 0.021	0.211 ± 0.007	0.152 ± 0.022
A12, C12, E12, G12	2048	0	0.470 ± 0.013	0.494 ± 0.010	0.473 ± 0.014	0.439 ± 0.020
B12, D12, F12, H12	2048	20	0.472 ± 0.010	0.320 ± 0.008	0.245 ± 0.035	0.161 ± 0.015

Appendix Table S5 - Cell growth in the 24 chemical environments used for library-scale measurement of LacI dose-response curves.

Cultures were grown at 37 °C with 0.5 mL culture volume per well and double-orbital shaking at 807 cycles per minute (see Materials and Methods for details). The final OD_{600} values were calculated by subtracting the offset from the gas-permeable membrane used during growth and are given as the mean ± standard deviation across the four wells used for each chemical environment. The time intervals between the starts of the incubation cycles for sequential growth plates was 10043 s, 10045 s, and 10038 s.

*Tetracycline was only added to Growth Plates 2-4.

region	start	first 12 bases	end	last 12 bases	nominal length	mutation rate
barcode 1	116	NNTNNNANNTNN	152	NNANNTNNNANN	37	N/A
inter-barcode	153	ATATGCCAGCAG	176	GCCGGCCACGCT	24	0.132
barcode 2	177	NNTNNNANNTNN	213	NNANNTNNNANN	37	N/A
intergenic	214	CGGTGGCCCGGG	394	CCTCCTGGATTA	181	0.352
lacI	395	TCACTGCCCGCT	1477	TACTGGTTTCAT	1083	6.478
regulatory	1478	ATTCACCACCCT	1789	GAGAGCTGCTAC	312	0.171
tetA	1790	ATGAGTAGCAGT	2992	GAAACGAGTGCC	1203	0.018
YFP	2993	TAACGGCGTAAG	3735	CTGTATAAATAA	743	0.021
KAN	3736	AAGCGGGAGACC	4779	GCGTCAGACCCC	1044	0.008
origin of replication	4780	TTAATAAGATGA	5584	TGCCAACATAGT	805	0.012
origin + intergenic	5585	AAGCCAGTATAC	115	GGCTGTCGGCGT	192	0.181

Appendix Table S6 - Regions extracted from long-read sequencing data for library-scale analysis.

The lacI region includes the *lacI* coding DNA sequence (CDS) and stop codon. The regulatory region includes the P_{lacI} and P_{tacI} promoters, the *lacO* operator, the riboJ insulator, and the RBS sites for both *lacI* and *tetA*. The tetA region includes the tetA CDS. The YFP region includes the YFP CDS and its RBS. The KAN region includes the transcriptional promoter and CDS for the kanamycin resistance gene as well as the transcriptional terminator for the genes regulated by LacI ($T_{L3S3P21}$). The origin of replication was split by the start of the PacBio circular consensus HiFi reads, so the last 32 bases of the origin of replication were grouped with the intergenic region between the origin and the barcodes. The start and end columns in the table give the nucleotide position of the start and end of each region based on the nominal full plasmid (GenBank ID: MT702633). The mutation rate column lists the estimated mean number of single nucleotide changes per 1000 bases for each region, using the nominal/wild-type sequence as the reference, and considers only single nucleotide polymorphisms (i.e. no indels). Regions adjacent to the barcodes and the lacI region have elevated mutation rates, due to the methods used for library generation and assembly.

Column	Nucleotide Sequence
DT.01	ATGGCGGCCGCTAGGGCCGGCGCGCCATCGAATGGCGCAAAACCTTTCGCGGTATGGCATGATAGCGCCCGGAAGAGAGTCAATTCAGGGTGGTGAAT
DT.02	CAGACGCGCCGAGACAGAACTTAATGGGCCC
DT.03	ACGCCGACAGCCCGGGCTCACCGATGGGGAAGACTAG
DT.04	AGCGGCCGCCATAAATCTCGAGCCAGTATACACTCCGCTA
DT.05	CGATGGCGCGCCGGCCCTAGCGGCCGCCATAAATCTCGAG
DT.06	GAGCCCGGGCTGTCGGCGTNNTNNNANNTNNNANNTNNNANNTNNNANNTNNNANNATATGCCAGCAGGCCGGCCGTGAGCTAACTCACATTAATTGCG
DT.07	GCTGTTAGCGGGCCCATTAAGTTCTGTCTCGGCGCGTCTGTTAAGCCAGCCCCGAC
DT.08	CGGTGGCCCGGGCGGCCGCACGATGCGTCCGGCGTAGAG
DT.09	TAATCCAGGAGGAAAAAATAATTGGTAACGAATCAGACAA
DT.10	TTGTCTGATTCGTTACCAATTATTTTTTCCTCCTGGATTA
DT.11	TCATTAGGCCTGTTTGGCCAGGCGCGCTGTTAGCGGGCCC
DT.12	CATCGTGCGGCCGCCCGGGCCACCGNNTNNNANNTNNNANNTNNNTNNTNNNANNTNNNANNAGCGTGGCCGGCCTTAAGCCAGCCCCGACACCCG
DT.13	ACTTAATGGGCCCGCTAACAGCGCGCCTGGCCAAACAGGCCTAATGATCTTCCGCTTCCTCGCTCACTG
DT.14	GTAAGCGGATGCCGGGAGCAGACAAGC
DT.15	CAAGAGCTACCAACTCTTTTTCCGAAGGTAACTGGCTTC

Appendix Table S7 – PCR primers used during LacI library construction

Sequences of primers used for PCRs during library construction.

Row	Nucleotide Sequence
A	ACACTCTTTCCCTACACGACGCTCTTCCGATCTNNNNNNNN**CGTGTATCTT**CATCGGTGAGCCCGGGCTGT
B	ACACTCTTTCCCTACACGACGCTCTTCCGATCTNNNNNNNN**ATTCATTGCA**CATCGGTGAGCCCGGGCTGT
C	ACACTCTTTCCCTACACGACGCTCTTCCGATCTNNNNNNNN**TCCTTCATAG**CATCGGTGAGCCCGGGCTGT
D	ACACTCTTTCCCTACACGACGCTCTTCCGATCTNNNNNNNN**GAATGCACGA**CATCGGTGAGCCCGGGCTGT
E	ACACTCTTTCCCTACACGACGCTCTTCCGATCTNNNNNNNN**GGAATTGTTC**CATCGGTGAGCCCGGGCTGT
F	ACACTCTTTCCCTACACGACGCTCTTCCGATCTNNNNNNNN**CCGGACCACA**CATCGGTGAGCCCGGGCTGT
G	ACACTCTTTCCCTACACGACGCTCTTCCGATCTNNNNNNNN**GACTTAGAAG**CATCGGTGAGCCCGGGCTGT
H	ACACTCTTTCCCTACACGACGCTCTTCCGATCTNNNNNNNN**TCTAGTCTTC**CATCGGTGAGCCCGGGCTGT

Appendix Table S8 – Forward index primers for barcode amplification.

Forward primer sequences used to amplify DNA barcodes from the plasmids. Underlined section anneals to plasmid. Bold section is the multiplexing tag sequence. N's are randomized positions used as unique molecular identifiers to correct for PCR jackpotting.

Column	Nucleotide Sequence
1	CTCGGCATTCCTGCTGAACCGCTCTTCCGATCTNNNNNNNN**TAGAGTTGGA**CCTCTACGCCGGACGCATCGT
2	CTCGGCATTCCTGCTGAACCGCTCTTCCGATCTNNNNNNNN**AGAGCACTAG**CCTCTACGCCGGACGCATCGT
3	CTCGGCATTCCTGCTGAACCGCTCTTCCGATCTNNNNNNNN**ACTCTACAGG**CCTCTACGCCGGACGCATCGT
4	CTCGGCATTCCTGCTGAACCGCTCTTCCGATCTNNNNNNNN**CGGTGACACC**CCTCTACGCCGGACGCATCGT
5	CTCGGCATTCCTGCTGAACCGCTCTTCCGATCTNNNNNNNN**GCGTTGGTAT**CCTCTACGCCGGACGCATCGT
6	CTCGGCATTCCTGCTGAACCGCTCTTCCGATCTNNNNNNNN**TGTGCTAACA**CCTCTACGCCGGACGCATCGT
7	CTCGGCATTCCTGCTGAACCGCTCTTCCGATCTNNNNNNNN**CCAGAAGTAA**CCTCTACGCCGGACGCATCGT
8	CTCGGCATTCCTGCTGAACCGCTCTTCCGATCTNNNNNNNN**CTTATACCTG**CCTCTACGCCGGACGCATCGT
9	CTCGGCATTCCTGCTGAACCGCTCTTCCGATCTNNNNNNNN**ACTAGAACTT**CCTCTACGCCGGACGCATCGT
10	CTCGGCATTCCTGCTGAACCGCTCTTCCGATCTNNNNNNNN**TTAGGCTTA**CCCTCTACGCCGGACGCATCGT
11	CTCGGCATTCCTGCTGAACCGCTCTTCCGATCTNNNNNNNN**TATCATGAGA**CCTCTACGCCGGACGCATCGT
12	CTCGGCATTCCTGCTGAACCGCTCTTCCGATCTNNNNNNNN**CTCACACAAG**CCTCTACGCCGGACGCATCGT

Appendix Table S9 - Reverse index primers for barcode amplification.

Reverse primer sequences used to amplify DNA barcodes from the plasmids. Underlined section anneals to plasmid. Bold section is the multiplexing tag sequence. N's are randomized positions used as unique molecular identifiers to correct for PCR jackpotting.

Column	Nucleotide Sequence
Primer F	AATGATACGGCGACCACCGAGATCTACACTCTTTCCCTACACGACGCTCTTCCGATCT
Primer R	CAAGCAGAAGACGGCATACGAGATCGGTCTCGGCATTCCTGCTGAACCGCTCTTCCGATCT

Appendix Table S10 - Primers sequences for the second PCR.

Primers used in the second PCR (15-cycle) to attach the standard Illumina paired-end adapter sequences and to amplify the resulting amplicons for sequencing. The underlined section anneals to the barcode amplification primers.

References

1. Markiewicz, P., Kleina, L. G., Cruz, C., Ehret, S. & Miller, J. H. Genetic Studies of the lac Repressor. XIV. Analysis of 4000 Altered Escherichia coli lac Repressors Reveals Essential and Non-essential Residues, as well as 'Spacers' which do not Require a Specific Sequence. *Journal of Molecular Biology* **240**, 421–433 (1994).
2. Pace, H. C. *et al.* Lac repressor genetic map in real space. *Trends in Biochemical Sciences* **22**, 334–339 (1997).
3. Lewis, M. *et al.* Crystal structure of the lactose operon repressor and its complexes with DNA and inducer. *Science* **271**, 1247–1254 (1996).
4. Swint-Kruse, L., Zhan, H., Fairbanks, B. M., Maheshwari, A. & Matthews, K. S. Perturbation from a distance: mutations that alter LacI function through long-range effects. *Biochemistry* **42**, 14004–14016 (2003).
